# Detectable call types in short-term passive acoustic monitoring of Atlantic forest and Caatinga primates

**DOI:** 10.1007/s10329-026-01280-1

**Published:** 2026-07-20

**Authors:** Juliana Carneiro de Lacerda, Ana Luiza L. Matte, Geovana Lima, Rick Taynor, Marcos Araújo, Bruna Marcela Teixeira de Andrade, Bárbara Lins Caldas de Moraes, Bruna Bezerra

**Affiliations:** 1 Programa de Pós-Graduação em Biologia Animal, Departamento de Zoologia, Laboratório de Ecologia, Comportamento e Conservação, Universidade Federal de Pernambuco, Av. Prof. Moraes Rego, 1235 - Cidade Universitária, Recife, PE 50670-901 Brazil; 2 Instituto SOS Caatinga, Povoado de Caboclo, São José da Tapera, AL 57445-000 Brazil

**Keywords:** Primate vocalisation, Acoustic activity, Acoustic signals, Vocal communication, Vocal repertoire

## Abstract

Arboreal primates rely heavily on acoustic communication, making them ideal candidates for passive acoustic monitoring (PAM). Here, we investigated the detectability of vocalisations from *Alouatta belzebul*, *Callithrix jacchus*, *Saimiri* spp, and *Sapajus flavius* in three fragments of Atlantic Forest and two fragments of Caatinga Forest in northeastern Brazil. We recorded each area for 48 h per month over 7 months, placing one audiomoth recorder at each site. Our results show that all species were detectable through PAM, with varying detection rates: 8/13 known call types for *C. jacchus*, 14/31 for *S. flavius*, 2/4 for *A. belzebul*, and 6/27 for *Saimiri* spp. Not all calls from their vocal repertoire were detected. Call detectability was influenced by species, habitat, time of day, and month. We emphasise the importance of considering behavioural, dietary, and home-range patterns when designing PAM protocols for primates. Our findings provide valuable insights for optimising PAM studies and highlight the need for continued basic research on vocal repertoire descriptions to improve conservation efforts.

**Supplementary Information:**

The online version contains supplementary material available at https://doi.org/10.1007/s10329-026-01280-1.

## Introduction

The use of Passive Acoustic Monitoring (PAM) has proven to be an effective tool for monitoring primate populations in various ecosystems, including the Atlantic Forest (e.g., *Callithrix penicillata*- Bittencourt et al. 2023; *Leontopithecus chrysopygus*- Manzano et al. 2023; Zambolli et al. 2023), Pantanal (*Alouatta macconnelli* - Do Nascimento et al. 2021; *Alouatta caraya*- Pérez-Granados and Schuchmann 2021), and Cerrado (*Sapajus libidinosus* -Lisboa et al. 2021). This is likely because many primate species produce loud vocalisations with specific characteristics adapted for long-distance transmission (Schneider et al. 2008). However, the acoustic properties of different environments, such as background noise, attenuation, amplitude fluctuation, and reverberation, can influence the structure and production of primate vocalisations (Brown and Waser 2017). To mitigate these effects, primates have evolved strategies to optimise their vocalisations, such as using lower frequency calls to reduce attenuation and increase propagation distance (Waser and Waser 1977).

Primates adapt their vocalisations to avoid masking by ambient noise, which can impact their ability to detect predators, hear alarm signals, and recognise conspecifics (Brown and Waser 2017). To mitigate this, they may adjust the timing and frequency of their vocalisations to coincide with periods of lower acoustic activity, as observed in species such as *Cercopithecus mitis*, *Lophocebus albigena*, *Cercopithecus aethiops*, and *Papio cynocephalus* (Brown et al. 1995; Waser and Brown 1986; Waser and Waser 1977). The primary sources of ambient noise, including biotic sound sources, wind noise, and vegetation movement, vary across habitats and impact sound propagation differently (Ey and Fischer 2009). Additionally, home range size can influence the production and transmission of acoustic signals, with primates in larger ranges often using lower frequency calls to improve transmission distance (Hunter and Krebs 1979; Mitani and Stuht 1998; Zimmermann 1995; Wich and Nunn 2002).

This study investigated the detectability of vocalisations from *Alouatta belzebul*, *Callithrix jacchus*, *Saimiri* spp., and *Sapajus flavius* in fragments of Atlantic and Caatinga Forests in northeastern Brazil using passive acoustic monitoring. Specifically, we examined how time of day, month, and call features (frequency and time parameters) may influence the acoustic detectability of those species. The Atlantic Forest is characterised by high primary productivity and a more continuous supply of primate food resources (e.g., fleshy fruits and insects) throughout the year (Morellato et al. 2000; Rothman et al. 2014). In contrast, the Caatinga is a semi-arid, seasonal forest where the availability of primate food resources is highly pulse-driven, with severe bottlenecks during the dry season (Leal et al. 2005). These environmental disparities may force primates to adopt different foraging and dispersal strategies to survive.

To understand how these environmental differences interact with primate biology, this study focuses on four sympatric species: *Callithrix jacchus*, *Sapajus flavius*, *Alouatta belzebul*, and *Saimiri* spp. These species exhibit varied social structures, body masses, and home range requirements (Table 1), factors that can directly modulate primate vocal behaviour and frequencies, potentially affecting their acoustic detectability in fragmented landscapes. By identifying key vocalisations detectable via PAM and potential factors affecting their detection, this study aims to provide valuable insights to inform and facilitate future primate surveys and experimental designs, ultimately contributing to more effective conservation and primate survey efforts.

**Table 1:** Summary of natural history and ecological traits of the target primate species

Species	Body Mass (references)	Group Size (references)	Population Density (references)	Home Range (references)
*Callithrix jacchus*	♀ 322.0 ± 40.4 g ♂ 317.9 ± 34.7 g (Araújo et al. 2000)	4–15 individuals (Stevenson and Rylands 1988)	9.5 ind./km² (Fialho and Gonçalves 2008)	~ 0.7–5.0 ha (Castro 2003; Francisco et al. 2015)
*Sapajus flavius*	♀ 2.02 ± 0.23 kg ♂ 2.74 ± 0.47 kg (ICMBio/CPB, unpublished data; Valença-Montenegro 2011)	Up to 160 individuals (Andrade et al. 2020)	0.11–0.50 ind./ha (Rodrigues et al. 2010)	~ 240 ha (Freire–Filho 2018)
*Alouatta belzebul*	♀ 3.02 (2.2–3.6) kg ♂ 4.57 (4.0–5.2) kg ICMBio/CPB, unpublished data; Valença-Montenegro et al. 2024	4–16 individuals (Silva 2022)	3.2 groups/km² (Silva 2022; Santos and Buss 2016)	4.8 ha − 10.6 ha (Ludwig et al. 2020; Coutinho 2012)
*Saimiri spp.*	♀ 635 g ♂ 740 g (Silvia Júnior et al. 2021)	Up to 130 individuals in large areas (Oliveira and Grelle 2012; Boinski 1999; Mendes-Pontes 1999; Camarotti et al. 2015; Campelo et al. 2019)	13.40 ind/km² (Mendes-Pontes, 1999)	9.2–130 há (Campelo et al. 2019)

## Methods

### Data collection

**Fig. 1:**
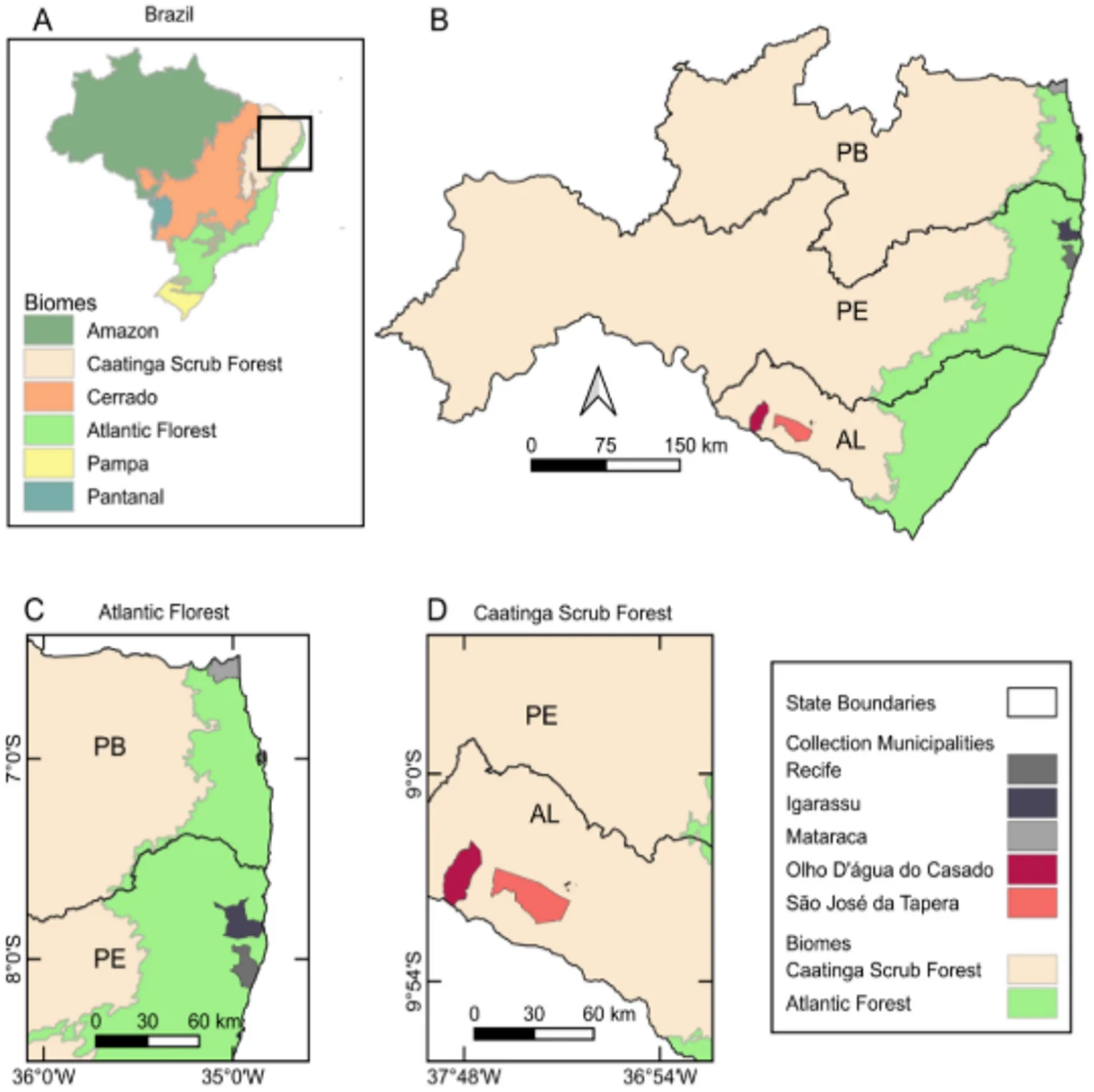
Location of collection sites. **A** Map of Brazil with biome boundaries; **B** Northeastern Brazilian states where we collected data (Paraíba, Pernambuco, and Alagoas); C) Data collection municipalities in the states of Paraíba (i.e., Mataraca; a 1,186 ha fragment) and Pernambuco (i.e., Igarassu, a 240 ha fragment; and Recife, a 106 ha fragment); D) Data collection in municipalities in the state of Alagoas (i.e., São José da Tapera, a 1,334.02 ha fragment; and Olho D’água do Casado, a 26,736.30 ha fragment)

We conducted Passive Acoustic Monitoring in three fragments of Atlantic Forest and two fragments of Caatinga Forest in northeastern Brazil, where the target species occur (Fig. 1; Supplementary material 1). An AudioMoth recorder (Open Acoustic Devices) was installed at one point in each of the five fragments. We recorded for 48 h each month for 7 months at the same location, totalling 336 h per study site. The selection of recording points in each site was based on previous primate detections in each sampled area. The recording units were positioned within the forest interior at varied distances from the forest edge because of the configuration of each fragment: 525 m in Mata da Tronox, Mataraca-PB (Area 1), 262 m in Mata do Curado Wildlife Refuge, Recife-PE (Area 2), 327.6 m in RPPN Morro da Torre, São José da Tapera-AL (Area 3), 81.7 m in MONA, Olho D’água do Casado-AL (Area 4), and 427 m in Mata dos Macacos, Igarassu-PE (Area 5). The recorders were programmed to continuously record for 48 h in each fragment at 16-bit, 48 kHz sampling rates, and to generate 1-hour sound files (WAV format) to facilitate analysis. Data collection occurred between September and October/2020; February and June/2022; February and August/2022; September/2022 and March/2023; July/2022 and January/2023. Data on study site characteristics, including climatic information, are available in Supplementary Material 1.

### Data analysis

We analysed the recordings using Kaleidoscope Pro version 5.4.2 (Wildlife Acoustic Inc.) software. The analysis involved grouping the recordings into clusters of different sounds and visually inspecting the spectrograms. Primate vocalisations were then selected and classified according to species. To identify primate species and vocalisation types, we followed established call classification systems for the targeted species: Bezerra and Souto (2008) and Epple (1968) for *Callithrix jacchus*, Oliveira (2002) for *Alouatta belzebul*, Bastos et al. (2015, 2018, 2019) for *Sapajus flavius*, and Coe and Rosenblum (2012) for *Saimiri* spp. We recorded the timing of vocalisations and classified them based on physical characteristics (frequency and temporal parameters). Chi-squared tests were used to investigate variations in acoustic detection throughout the day (04:00–06:00, 07:00–09:00, 10:00–12:00, 13:00–15:00, and 16:00–18:00) and months. We also estimated the anthropogenic noise rate across the study sites by counting detections of man-made noise during the 48 h recording periods. We then estimated the per-hour man-made noise detection.

## Results

### Types of vocalisations detectable by PAM

**Fig. 2:**
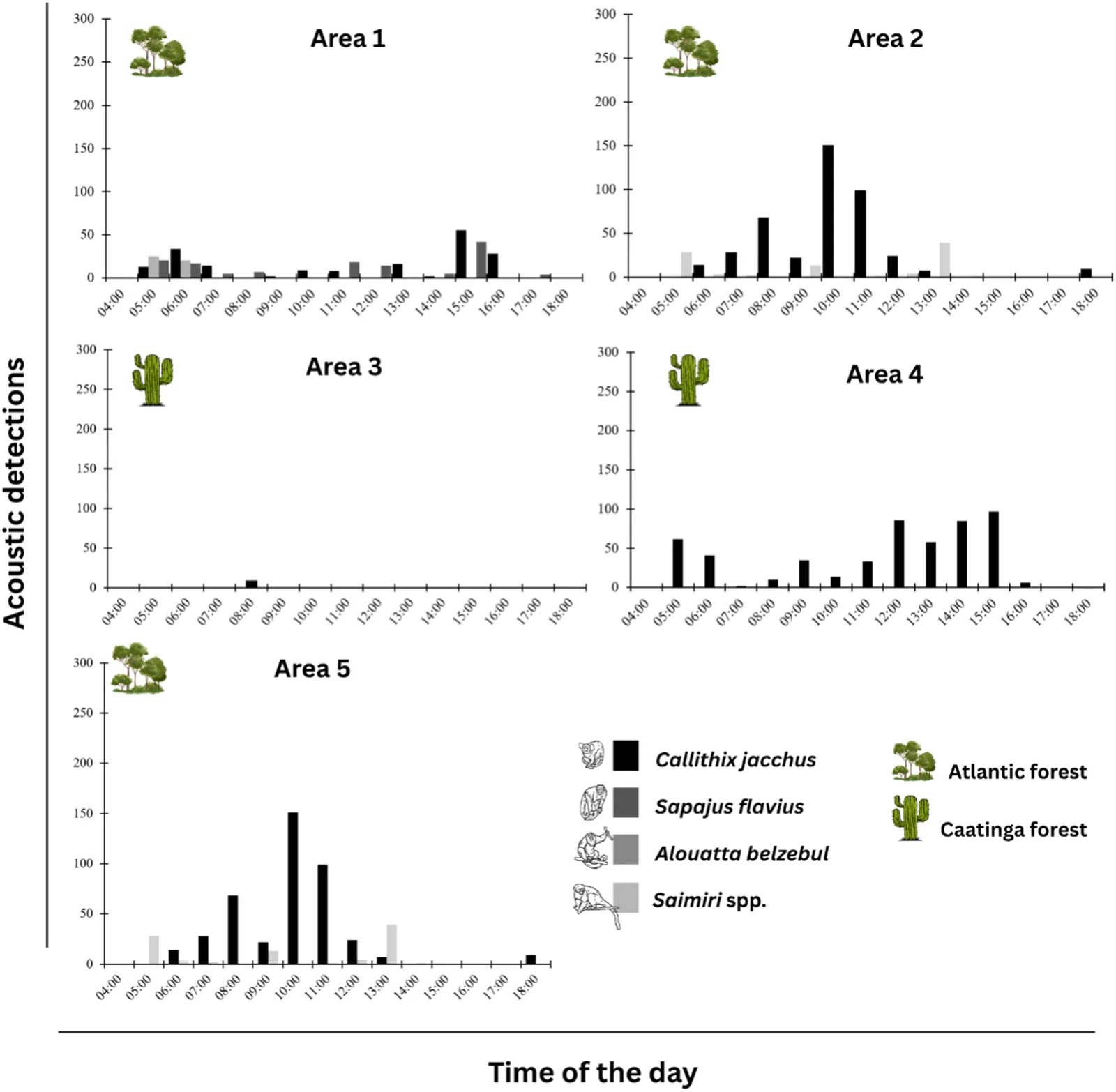
Detections of the species *Callithrix jacchus*, *Sapajus flavius*, *Alouatta belzebul*, and *Saimiri* spp. throughout the day in the Atlantic Forest and Caatinga Forest fragments analysed in the study. Area 1: Mata da Tronox, Mataraca-PB (1186 ha); Area 2- Mata do Curado Wildlife Refuge, Recife-PE (106 ha); Area 3-RPPN Morro da Torre, São José da Tapera-AL (1334.02 ha); Area 4 - MONA, Olho D’água do Casado-AL (26736.30 ha); Area 5- Mata dos Macacos, Igarassu-PE (240 ha)

We obtained 3177 records of primate vocalisations through 1680 h of PAM. Long-distance *phee* calls of *Callithrix jacchus* were the most frequently detected call types across all sites. Not all known call types from each species were detectable (Supplementary material 2). We found variations in the detection of primate vocalisation throughout the day (Table 2; Fig. 2) and months (Table 3). Primate vocal detection patterns varied across study sites (Fig. 2). In Area 1, *Callithrix jacchus* detections were concentrated in the early morning (between 05:00 and 07:00) and late afternoon (15:00 and 16:00), while detection of *Sapajus. flavius* showed a marked peak in the early afternoon between 13:00 and 15:00. *Alouatta belzebul* vocalizations were more frequently detected in the early morning (05:00). In Area 2, *Callithrix jacchus* showed a distinct detection peak around 10:00, and *Saimiri* spp, showed an increase in detection in the early afternoon (13:00). Area 3 exhibited low vocal detection, with only eight *Callithrix jacchus* detections concentrated at 8:00. In Area 4, *Callithrix jacchus* exhibited a pattern with detection peaks at 05:00 and 10:00. In Area 5, detections of *Callithrix jacchus* were distributed throughout the day. *Sapajus flavius* showed a peak in detection around 13:00 (Fig. 2). The anthropogenic noise rates across the study site ranged from 0.31 to 0.87 detections/hour (supplementary material 1).

**Table 2:** Comparison of the detection of primate vocalisations between the different blocks of daylight hours (i.e., 4:00–6:00; 7:00–9:00; 10:00–12:00; 13:00–15:00; 16:00–18:00)

Area	Biome	Species	Diurnal variation (Chi-Square Tests), DF = 4
1	*Atlantic Forest*	*Callithrix jacchus*	X^2^: 64; *p* < 0.001
		*Sapajus flavius*	X^2^: 46.36; *p* < 0.001
		*Alouatta belzebul*	X^2^: 1936; *p* < 0.001
2	*Atlantic Forest*	*Callithrix jacchus*	X^2^: 633; *p* < 0.001
		*Saimiri* spp.	X^2^: 45.25; *p* < 0.001
3	*Caatinga Forest*	*Callithrix jacchus*	X^2^: 64; *p* < 0.001
4	*Caatinga Forest*	*Callithrix jacchus*	X^2^: 304.11; *p* < 0.001
5	*Atlantic Forest*	*Callithrix jacchus*	X^2^: 783.94; *p* < 0.001
		*Sapajus flavius*	X^2^: 565.69; *p* < 0.001

Areas: 1 - Mata da Tronox, Mataraca-PB; 2 - Mata do Curado Wildlife Refuge, Recife - PE; 3 - RPPN Morro da Torre, São José da Tapera-AL; 4 - MONA, Olho D’água do Casado-AL; 5 - Mata dos Macacos, Igarassu-PE

**Table 3:** Variation in the detection of primate vocalisations during the collection months

Area	Biome	Species	Highest Detection Month	Average number of detections per month ± SD*	Monthly variation (Chi-Square Tests) DF = 6
1	*Atlantic Forest*	*Callithrix jacchus*	April (*n* = 95)	25.57 ± 32.28	*X*^2^: 244.64; *p* < 0.001
		*Sapajus flavius*	April (*n* = 84)	19 ± 30.54	*X*^2^:260879.63; *p* < 0.001
		*Alouatta belzebul*	February (*n* = 45)	6.42 ± 15.74	*X*^2^: 1936.00; *p* < 0.001
2	*Atlantic Forest*	*Callithrix jacchus*	May (*n* = 144)	60.57 ± 43.52	*X*^2^: 218.98; *p* < 0.001
		*Saimiri* spp.	July (*n* = 46)	13 ± 13.59	*X*^2^: 86.54; *p* < 0.001
3	*Caatinga Forest*	*Callithrix jacchus*	September (*n* = 9)	1.28 ± 3.14	*X*^2^: 64; *p* < 0.001
4	*Caatinga Forest*	*Callithrix jacchus*	January (*n* = 418)	75.57 ± 143.58	*X*^2^: 278,784; *p* < 0.001
5	*Atlantic Forest*	*Callithrix jacchus*	October (*n* = 327)	213.57 ± 122.18	*X*^2^:2232036.00; *p* < 0.001
		*Sapajus flavius*	October (*n* = 148)	50.28 ± 59.36	*X*^2^:123201.00; *p* < 0.001

Areas: 1 - Mata da Tronox, Mataraca-PB; 2 - Mata do Curado Wildlife Refuge, Recife - PE; 3 - RPPN Morro da Torre, São José da Tapera-AL; 4 - MONA, Olho D’água do Casado-AL; 5 - Mata dos Macacos, Igarassu-PE. *48 h of recordings in each month (*n* = 7 months)

## Discussion

We detected vocalisations of *Callithrix jacchus*, *Sapajus flavius*, *Saimiri* spp., and *Alouatta belzebul* through passive acoustic monitoring (PAM) in northeastern Brazil. Species identification in the recordings was facilitated by advances in basic research describing species-specific vocal repertoires. However, not all species were detected in all biomes, and not all known call types of each species were detectable. The time of day and month influenced primate call detection. Species-specific differences in detection suggest that distribution, social and feeding ecology, call characteristics, and propagation patterns influence PAM detectability.

Common marmosets’ vocalisations were the most frequently recorded across all areas, suggesting that species with smaller home ranges and more limited travelling behaviour are more likely to be detected by PAM. Studies suggest that efficiency of PAM detection of calls is influenced by primate displacement patterns (Pérez-Granados and Schuchmann 2021), which are shaped by space use, home range size, and behavioral and ecological factors, such as social aspects (Milton and May 1976), feeding strategies (Dietz et al. 1997; Peres 1994; Rylands 1996), suitable resting places (Altmann 1974; Hankerson et al. 2007), and forest canopy connectivity (Raboy et al. 2004). Diet type may also affect movement patterns, with food availability and abundance influencing home range size (Fleagle 1988). *Callithrix jacchus* has a flexible diet that includes fruits and insects, but it also specialises in feeding on widely available gum (Rylands et al. 1993; Power and Myers 2009). This dietary flexibility, combined with a relatively small home range (0.7–5.0 ha) and territorial behaviour, likely increases its likelihood of being detected consistently in PAM. When considering the combined data across the Atlantic Forest fragments, the high detectability of *Callithrix jacchus* remains consistent across different months, suggesting that seasonal fluctuations in resource availability may not strictly limit its acoustic detection. In the Atlantic Forests, *Callithrix jacchus* groups have been observed to expand their home range during rainy periods to exploit fluctuating fruit resources (Castro 2003). Conversely, during the dry season, when fruit and insect availability may decrease, groups rely on exudates (Castro et al. 2000). Since gum sources are often spatially concentrated, this dietary shift can lead to reduced travel distances and more localised activity. However, because gum is a reliable, year-round resource, *Callithrix jacchus* often maintains stable activity periods and group stability regardless of the season (Ferrari and Ferrari 1989). Therefore, our results suggest that despite seasonal shifts in some resource availability, the acoustic activity of *Callithrix jacchus* remains highly detectable by PAM throughout the year, reinforcing the method’s effectiveness for monitoring the species in both dry and wet months.

Blonde capuchins were detected only in Atlantic Forest fragments, despite their known occurrence in the Caatinga Forest study sites, suggesting differences in habitat use or recorder detectability. Blonde capuchins’ habitat adaptability is facilitated by an omnivorous diet (Bicca-Marques et al. 2006) and a fission-fusion social structure, which optimises space use and reduces competition (Medeiros et al. 2019). During periods of food scarcity, groups disperse, increasing movement and foraging effort (Izar 2004; Izar et al. 2012). The contrasting food availability between the Caatinga and Atlantic Forest likely requires capuchins to adjust their diets and exploit alternative resources. They may need to travel longer distances in the Caatinga forest than in the Atlantic Forest due to differences in resource availability, which could affect PAM detectability. The absence of acoustic detection of *Sapajus flavius* in Caatinga sites such as São José da Tapera and Olho D’água do Casado may be explained by the species’ relatively low population densities (0.11 to 0.33 ind/ha) (Rodrigues et al. 2010) and relatively large home ranges (up to 240 ha) (Filho 2018). These primates often adopt a fission-fusion social structure or wide-ranging foraging behaviour to cope with resource scarcity. Consequently, the probability of a group passing and vocalising within the effective detection radius of a single stationary recorder during a 48-hour session is low.

Differences in temperature and humidity between the Atlantic Forest and Caatinga (Braga and Laurini 2024) can potentially impact both recorder performance (but see Lapp et al. 2023) and primate vocal activity and propagation (Ferrario et al. 2024). However, our data showed that mean temperatures were relatively stable across biomes, with the Caatinga sites (25.4 °C) presenting lower average temperatures than some Atlantic Forest fragments (e.g., Area 1 at 26.6 °C). This indicates that temperature-induced equipment failure or thermal stress was likely not the main driver of the observed detectability patterns. The recorded temperatures (~ 25–26 °C) remained well within the optimal operating range for AudioMoth devices (Hill et al. 2019; Lapp et al. 2023). Furthermore, these values fall within the thermoneutral zone for neotropical primates, where vocal activity is not necessarily suppressed by heat (Campos and Fedigan 2009). On the other hand, relative humidity was consistently lower in the Caatinga (71.9%) than in the Atlantic Forest (up to 77.6%). From a physical standpoint, lower humidity reduces atmospheric absorption and facilitates the transmission of high-frequency signals (Harris 1966), such as some calls of *Callithrix jacchus* and *Sapajus flavius*. Therefore, the absence of *Sapajus flavius* detections at the Caatinga forest sites is more likely due to ecological factors, such as the species’ fission-fusion social structure and the wider-ranging foraging strategies required in semi-arid environments.

Despite its characteristic loud howling, *Alouatta belzebul* was the least detected species in our PAM survey using a single recording unit. This outcome contrasts with other studies that employed multiple recorders and were conducted in areas with larger *Alouatta* populations, which likely increased detection probabilities (e.g., Do Nascimento et al. 2021; Pérez-Granados and Schuchmann 2021). Notably, long-term monitoring of the study area (since 2010) indicates that *Alouatta belzebul* was first observed in February 2022, when two individuals were sighted, presumably dispersing from adjacent fragments (Bezerra, personal communication). Thus, the low detection rate for *Alouatta belzebul* in our PAM survey may be attributed, at least in part, to the small population size and relatively recent arrival of *A. belzebul* in the fragment. We highlight that *Alouatta belzebul* was not expected in the Caatinga forest sites because the species’ distribution in Alagoas state is restricted to Atlantic Forest remnants (Olímpio 2024).

Primate social and home range attributes (Table 1) and fragment sizes (Supplementary Material 1, Fig. 1) create an important trade-off for PAM success. The forest fragments in our study vary in size. In the Atlantic Forest, they ranged from 106 ha (Area 2) to 1,186 ha (Area 1). In the Caatinga forest, they ranged from 1,334.02 ha (Area 3) to 26,736 ha (Area 4). In Area 3 (São José da Tapera), although the fixed recorder was located within the 20-ha private reserve RPPN Morro da Torre, the area is part of a continuous forest covering 1,334.02 ha. For monkeys with large home ranges and fission-fusion organisations, such as *Sapajus flavius* (up to 240 ha; Table 1), a single fixed recorder per site creates a substantial spatial gap relative to the population’s potential day range (Pérez-Granados and Schuchmann 2021). In these massive landscapes, groups can easily forage far from the recorder to exploit seasonal agricultural resources (Lins and Ferreira 2019; Falotico and Ottoni 2023). This may help explain why *Sapajus flavius* went undetected in the Caatinga despite its known occurrence at both study sites. The device works for identifying their calls when the animals are relatively close, but the likelihood of detecting them is reduced when they are miles from the recording device during the 48-hour recording period (Hoefer et al. 2025; Kalan et al. 2015). To boost detection in these scenarios, deploying multiple devices across the site may be more efficient.

In addition, the distance between the recorder and the forest edge affects acoustic detections (Sugai et al. 2019; Pérez-Granados and Schuchmann 2021). In the Area 4 fragment (i.e., *Morro dos Macacos*), we installed the recorder near the São Francisco River, just 81.7 m from its edge. This proximity may explain the relatively high rate of boat noise recorded (0.87 sounds per hour; Table 2). Open river areas allow low-frequency boat engine noise to propagate easily into the vegetation (Marten and Marler 1977; Wiley and Richards 1978). This constant mechanical noise can cause stress in animals or disrupt communication (De Almeida-Rocha et al. 2017; Duarte et al. 2018), and, thus, may contribute to the lack of detection of *Sapajus flavius* at the sites. In contrast, in forests where the recorders were placed farther from the forest edge, such as Area 2 (262 m), Area 3 (327.6 m), Area 5 (427 m), and Area 1 (525 m), dense vegetation may help block man-made noise. This quieter environment allows the recorder to continuously detect abundant species, such as *Callithrix jacchus*.

A closer look at the monthly data in Table 2 reveals a mixed pattern across sites rather than a clear seasonal trend. This lack of a uniform pattern may arise because both weather conditions and primates’ behaviours change constantly. From a physical standpoint, lower humidity reduces sound absorption in the air, which theoretically helps the recorder detect calls from farther away (Harris 1966). Nevertheless, these physical advantages can be cancelled out by local changes in human noise or by the primates’ daily movements (Bradbury and Vehrencamp 1998; Sugai et al. 2019). From a biological standpoint, as shown in Table 1, species with large home ranges and fission-fusion organisations (such as *Sapajus flavius*) or flexible diets (such as *Callithrix jacchus*) can change their daily paths (Izar et al. 2012; Rylands et al. 1993; Power and Myers 2009). With a sampling design of 48 h per month per recorder, a high number of detections in a given month most likely indicates that a foraging group spent time near the recorder by chance, rather than a true seasonal peak (Heinicke et al. 2015). Understanding that short-term PAM captures accidental encounters, depending on the species, rather than predictable seasonal curves, is essential for correctly interpreting how primates use fragmented landscapes (Heinicke et al. 2015; Pérez-Granados and Schuchmann 2021).

Regarding *Saimiri spp.*, their detection in the Atlantic Forest remnants is likely favoured by their large social groups (over 100 individuals) and high vocal activity (Campelo et al. 2019), which increases the likelihood of detecting signals even in fragmented landscapes where they have been introduced. Furthermore, the frequent use of long-distance vocalisations used to maintain group cohesion may have enhanced PAM efficiency for this taxon. The high vocal activity and the use of these far-reaching signals suggest that even in introduced populations, species-specific vocal traits are key determinants of acoustic monitoring success.

Detected calls spanned 1.2–17.3 kHz, with optimal detection between 8 and 9 kHz and durations of 300–1300 ms, aligning with *Callithrix jacchus*’ long-distance Phee calls. Anthropogenic noise likely influenced the propagation of vocalisations, as it was present across all study areas (see supplementary material 1), potentially prompting animals to modulate their calls for optimal transmission. In noisy environments, animals may alter their vocalisation rate, thereby reducing energy costs to maintain group cohesion (Bittencourt et al. 2023). The high rate of anthropogenic noise in Area 4 (0.87 detections/hour) suggests a compensatory vocal mechanism to ensure signal transmission or an increased alert response to human disturbance (Brumm and Slabberkoorn 2005). This modulation is consistent with uneven acoustic signal propagation, prompting animals to adjust vocalisations (Richards and Wiley 1980). Anthropogenic noise can also lead to temporal changes in activity patterns, resulting, for instance, in nocturnal activity in diurnal species in environments with greater anthropogenic disturbance (Spaan et al. 2025; Donati et al. 2016). The diurnal *Callithrix jacchus* also showed some vocal activity when it was already dark in our study (between 18 h and 19 h), supporting this notion.

In conclusion, our study provides insights into the vocalisations most likely to be detected for the targeted primate species using PAM, emphasising the importance of basic research on vocal repertoires to inform and optimise passive acoustic monitoring protocols. Effective PAM design for primates requires consideration of key factors, including group size, social and activity patterns, home range, vocalisation structure, space use, and resource availability. By integrating these parameters, the accuracy and efficiency of future PAM studies may be enhanced, ultimately contributing to improved conservation and survey efforts for primate populations.

## Supplementary Information

Below is the link to the electronic supplementary material.

Supplementary Material 1

Supplementary Material 2

## Data Availability

Please contact the corresponding author for information.
