# Supplementary material for: Detectable call types in short-term passive acoustic monitoring of Atlantic forest and Caatinga primates
Source: Primates. 2026 Jul 20;67(6):745–54. doi: 10.1007/s10329-026-01280-1 (PMC13731453; doi:10.1007/s10329-026-01280-1)
Supplement: Supplementary file 1 [file 10329_2026_1280_MOESM1_ESM.docx]

**Supplementary Material 2**: Call types detected through Passive acoustic monitoring of the study species in fragments of Atlantic Forest and Caatinga forest in Northeast Brazil.

| **Species** | **Home-range size** | **Number of call types described/number of call types detected** | **Frequency range of vocalizations described in the vocal repertoire** | **Call types detected** | **Sources for vocal repertoire* and home range size**** |
| --- | --- | --- | --- | --- | --- |
| *Callithrix jacchus* | 5.6 ha | 13 /7 | Long Phee Call (8.80 kHz - 7.07 kHz)  Brief Phee Call 1 (8.91 kHz - 7.24 kHz)  Brief Phee Call 2 (10.06 kHz - 7.65 kHz)  Brief Phee Call 3 (8.61 kHz - 7.47 kHz)  Tisik Call (14,63 kHz - 2.72 kHz)  Very Brief Whistle (17.39 kHz - 14.68 kHz)  Twitter Call (13.08 kHz - 5.49 kHz) | Long Phee Call (n=1982)  Brief Phee Call 1 (n=345)  Brief Phee Call 2 (n=77)  Brief Phee Call 3 (n=66)  Tisik Call (n=8)  Very Brief Whistle (n=5)  Twitter Call (n=1). | Bezerra and Souto 2008*;  Pinheiro et al. 2015.** |
| *Sapajus flavius* | 240,22 ha | 29/14 | Huh (1.01 kHz - 0.71 kHz)  Huh-3var (1.51 kHz - 1.09 kHz)  Lost Call (1.49 kHz - 1.11 kHz)  Howl (1.66 kHz - 1.14 kHz)  Fear Call (2.15 kHz - 1.63 kHz)  Huh-1var (1.33 kHz - 1.07 kHz)  Hoot (4.59 kHz - 1.94 kHz)  Bellow (10.68 kHz - 0.53 kHz)  Huh-4var (2.32 kHz - 1.93 kHz)  Shout (no information)  Clack (10.11 kHz - 1.28 kHz)  Heh (4.5 kHz - 0 kHz)  Huh-2var (2.0 kHz - 1.46 kHz)  Cheep (3.05 kHz - 2.0 kHz) | Huh (n=91)  Huh-3var (n=56)  Lost Call (n=51)  Howl (n=37)  Fear Call (n=29)  Huh-1var (n=25)  Hoot (n=20)  Bellow (n=6)  Huh-4var (n=5)  Shout (n=5)  Clack (n=4)  Heh (n=3)  Huh-2var (n=2)  Cheep (n=1). | Bastos et al. 2015 * Rodrigues K. C. 2013** |
| *Alouatta belzebul* | 17.8 ha | 6/2 | Short Roar (*Alouatta fusca clamitans* (0.95 kHz - 1.2 kHz)  Roar (0.4 kHz - 1.6 kHz) | Short roar (n=4)  Roar (n=41) | Oliveira, D. G. 2002*  Pinto et al.2003** |
| *Saimiri* spp. | 75.0 – 77.5 ha | 26/6 | Isolation Peep (12.0 kHz - 9.0 kHz)  Location Trill (9.0 kHz - 6.0 kHz)  Peep Calls (5 kHz - 16 kHz)  Roman Chucks (2 kHz - 5 kHz) | Isolation Peep (n=32) Location Trill (n=20)  Short Peep (n=19)  Ascending Peep (n=18)  Play Peep (n=1)  Roman Chucks (n=1) | Hammerschmidt et al. 2001*  Winter et al. 1966*  Pinheiro et al. 2013** |

**References**

Bastos M, Souto A, Jones G, Eason P, Bione C, Schiel N, and Bezerra B (2015) Vocal repertoire of wild blonde capuchins (*Sapajus flavius*) and contextual use of calls. Am J Primatol 77(6): 605–617.

Bezerra BM, and Souto A (2008) Structure and usage of the vocal repertoire of *Callithrix jacchus*. Int J Primatol 29: 671-701.

Hammerschmidt K, Freudenstein T, and Jürgens U (2001) Vocal development in squirrel monkeys. Behaviour 138(9), 1179-1204.

Oliveira DAG (2002 Vocalizações de longo alcance de *Alouatta fusca clamitans* e *Alouatta belzebul belzebul*: estrutura e contextos. Tese (Doutorado), Instituto de Psicologia - Universidade de São Paulo.

Pinheiro HLN, and Mendes Pontes AR (2015) Home range, diet, and activity patterns of common marmosets (*Callithrix jacchus*) in very small and isolated fragments of the Atlantic Forest of northeastern Brazil. Int J Ecol 2015(1), 685816.

Pinto AC, Azevedo-Ramos C, and De Carvalho Jr O (2003) Activity patterns and diet of the howler monkey *Alouatta belzebul* in areas of logged and unlogged forest in Eastern Amazonia. Anim Biodivers Conserv 26(2), 39-49.

Rodrigues KC (2013) Padrão de atividades, comportamento alimentar, exploração de habitat e área de vida de um grupo de *Sapajus flavius* (Schreber, 1774) (Primates, Cebidae) em um fragmento de floresta atlântica, Paraíba, Brasil.

Winter P, Ploog D, and Latta J. 1966. Vocal repertoire of the squirrel monkey (*Saimiri sciureus*), its analysis and significance. Exp Brain Res 1(4), 359-384.
