# Supplementary material for: Detectable call types in short-term passive acoustic monitoring of Atlantic forest and Caatinga primates
Source: Primates. 2026 Jul 20;67(6):745–54. doi: 10.1007/s10329-026-01280-1 (PMC13731453; doi:10.1007/s10329-026-01280-1)
Supplement: Supplementary file 2 [file 10329_2026_1280_MOESM2_ESM.docx]

**Supplementary material 1.**

Study site characteristics and recording period. PAM: Passive acoustic monitoring. *Climatic data (mean temperature and mean humidity) during the sampling periods were obtained from the Brazilian National Institute of Meteorology (INMET) and the Agrometeorological monitoring system (Agritempo). Due to the local availability of active stations, the mean temperature for Area 1 - Mataraca (Paraíba) was retrieved from the Agritempo database, while the mean humidity for the same site was obtained from the João Pessoa INMET station (approx. 85 km away). Data for Area 2 - Recife and Area 5 - Igarassu (Pernambuco) were retrieved from the Surubim station, and for Area 4 - Olho D’água do Casado and Area 3 - São José da Tapera (Alagoas), from the Piranhas station. These sources were selected as they provided the most complete and reliable datasets for the respective regions during the study period.

| Area | GPS Location | Municipality | Mean Humidity and range (%) * | Mean Temperature and range (C°) * | Protection Status | Primates occurring in the area | Number of records vocalizations through PAM | Biome | Noise Rate detection/h (key noise) | Recording Period | Brief description |
| --- | --- | --- | --- | --- | --- | --- | --- | --- | --- | --- | --- |
| 1 | 06°29.902‟S, 034º29.704‟W | Mataraca - PB | 77.6% (98% - 46%) | 26.6 C° (31 - 22 C°) | Private area – Mata da Tronox | *Alouatta belzebul*, *Callithrix jacchus*, *Sapajus flavius* | 45  181  133 | Atlantic Forest | 0.56 (Road traffic) | September to October 2020 and February to June 2022 | It has 1186 ha, consisting of a mosaic of different phytophysiognomies (areas of natural and reforested forest, mangrove, and floodplain - Medeiros et al., 2019) surrounded by a sugarcane matrix, a wind farm, and an urban matrix. |
| 2 | 8°04'50"S, 34°58'21"W | Recife - PE | 74.6 (94 - 30) | 24.2 (35 - 16.7) | Protected area / Mata do Curado Wildlife Refuge | *Saimiri spp*, *Callithrix jacchus* | 91  423 | Atlantic Forest | 0.47 (Road traffic) | February to August 2022 | It has 106 ha and is considered an urban forest fragment as it is located in the Metropolitan Region of Recife. |
| 3 | 9°33'13.34"S, 37°32'39.26"W | São José da Tapera- AL | 71.9 (98 - 27) | 25.4 (36.9 - 16.8) | Protected area / Morro da Torre Private Reserve | *Callithrix jacchus Sapajus Flavius* | 9  0 | Caatinga Scrub Forest | 0.31 (Domestic dog barks) | July 2022 to January 2023 | It covers 1,334.02 ha, from which 20 ha was granted protected area status in April 2023. |
| 4 | 9° 33′ 24″ S, 37° 52′ 3″ W | Olho D’água do Casado - AL | 71.9 (98 - 27) | 25.4 (36.9 - 16.8) | Protected area / São Francisco River Natural Monument (MONA) | *Callithrix jacchus Sapajus flavius* | 529  0 | Caatinga Scrub Forest | 0.87 (Boat engines) | July 2022 to January 2023 | MONA covers 26,736.30 hectares across five municipalities in the Alagoas hinterland. The data collection occurred in an area known as “Morro dos Macacos”. |
| 5 | 07°47.162‟S, 035°00.788‟W | Igarassu, -PE | 74.6 (94 - 30) | 24.2 (35 - 16.7) | Private area – Mata dos Macacos | *Callithrix jacchus, Sapajus flavius.* | 1419  360 | Atlantic Forest | 0.51 (Road traffic) | September 2022 to March 2023 | Mata dos Macacos is a 240 ha of dense ombrophilous forest set in a sugarcane matrix (Pereira, 2016). |
